# Phenotypic Overlap between MMP-13 and the Plasminogen Activation System during Wound Healing in Mice

**DOI:** 10.1371/journal.pone.0016954

**Published:** 2011-02-04

**Authors:** Anna Juncker-Jensen, Leif R. Lund

**Affiliations:** 1 Finsen Laboratory, Rigshospitalet, Copenhagen Biocenter, Copenhagen, Denmark; 2 Department of Cellular and Molecular Medicine, Faculty of Health Sciences, University of Copenhagen, Copenhagen, Denmark; Brigham and Women's Hospital, United States of America

## Abstract

**Background:**

Proteolytic degradation of extracellular matrix is a crucial step in the healing of incisional skin wounds. Thus, healing of skin wounds is delayed by either plasminogen-deficiency or by treatment with the broad-spectrum metalloproteinase (MP) inhibitor Galardin alone, while the two perturbations combined completely prevent wound healing. Both urokinase-type plasminogen activator and several matrix metallo proteinases (MMPs), such as MMP-3, -9 and -13, are expressed in the leading-edge keratinocytes of skin wounds, which may account for this phenotypic overlap between these classes of proteases.

**Methodology:**

To further test that hypothesis we generated *Mmp13;Plau* and *Mmp13;Plg* double-deficient mice in a cross between *Mmp13*- and *Plau*-deficient mice as well as *Mmp13*- and *Plg*-deficient mice. These mice were examined for normal physiology in a large cohort study and in a well-characterized skin wound healing model, in which we made incisional 20 mm-long full-thickness skin wounds.

**Principal Findings:**

While mice that are deficient in *Mmp13* have a mean healing time indistinguishable to wild-type mice, wound healing in both *Plau*- and *Plg*-deficient mice is significantly delayed. Histological analysis of healed wounds revealed a significant increase in keratin 10/14 immunoreactive layers of kerationcytes in the skin surface in *Mmp13;Plau* double-deficient mice. Furthermore, we observe, by immunohistological analysis, an aberrant angiogenic pattern during wound healing induced by *Plau*-deficiency, which has not previously been described.

**Conclusions:**

We demonstrate a phenotypic overlap, defined as an additional delay in wound healing in the double-deficient mice compared to the individual single-deficient mice, between MMP-13 and the plasminogen activation system in the process of wound healing, but not during gestation and in postnatal development. Thus, a dual targeting of uPA and MMP-13 might be a possible future strategy in designing therapies aimed at tissue repair or other pathological processes, such as cancer invasion, where proteolytic degradation is a hallmark.

## Introduction

Proteolytic degradation and remodeling of the extracellular matrix (ECM) is essential for several physiological and pathological processes, such as embryonic development, mammary gland morphogenesis, tissue remodeling and repair, cancer invasion and metastasis [1]–[7]. Matrix metalloproteinases (MMPs) are a family of 24 human extracellular zinc-dependent endopeptidases, some of which are capable of degrading a variety of ECM components. In addition, other substrates for MMPs include growth factors and cytokines and other proteases [8] (for a detailed review see [9]). MMP-13, or collagenase-3, is among the limited numbers of MMPs that can cleave the fibrillar collagens (I, II and III), being particularly potent against collagen II [10], the main collagen constituent in cartilage. The expression of MMP-13 is seen in numerous cancer types, particularly carcinomas [11], and it has been suggested that MMP-13 could be an indicator for the invasive capacity of squamous cell carcinomas (SCCs) [12], [13].

The plasminogen activation (PA) system of serine proteases is an additional extracellular proteolytic system involved in physiological and pathological tissue remodeling [14]. Plasmin, the main fibrinolytic enzyme in the body, is formed from plasminogen (Plg) by proteolytic cleavage by one of three Plg activators, the urokinase type (uPA), tissue type (tPA) [15] or plasma kallikrein [16]. While uPA converts Plg to plasmin within tissues, tPA generates plasmin for thrombolysis. Besides fibrin, other major substrates for plasmin include fibronectin, laminin, latent TGF-β1, pro-uPA and, at least *in vitro*, pro-MMPs including MMP-13 [17]. While *Plg*-deficiency has serious physiological consequences in both mice and humans [18]–[20], the effects of *Plau*- (the gene designation for uPA) deficiency are less pronounced [21]. However, numerous prognostic studies have correlated high uPA protein levels in tumor tissue and blood samples with poor prognosis in many types of cancers [14]. While uPA in some types of cancers is expressed by stromal cells such as fibroblasts and macrophages, uPA in SCCs is expressed by the cancer cells themselves [22]. Several components of the MMP and PA families have a very similar expression pattern in cancer invasion and certain types of non-neoplastic tissue-remodeling, such as wound healing. It has therefore been suggested that cancer invasion can be viewed as tissue remodeling gone out of control [23]–[25]. In both skin SCC and skin wound healing, epithelial cells (cancer cells and keratinocytes) express MMP-13 and uPA [22], [26]–[30] and in mice MMP-13 appears to be the main collagenase involved in epidermal keratinocyte migration across a collagen- and fibrin-rich provisional matrix in vitro [31].

Wound healing in skin involves three partially overlapping phases: inflammation, proliferation and tissue remodeling. During proliferation, keratinocytes migrate and hyperproliferate at the wound edge, leading to coverage of the wound with a new epidermis, a process called re-epithelialization [25], [32]. Different proteases have been implicated in the various phases of wound healing, with MMPs and serine proteases being the most important [33]. Mice deficient in *Plg* show severely impaired wound healing, due to a diminished ability of the migrating keratinocytes to dissect the fibrin-rich matrix [19], [34]. Time to complete wound healing in *Plau*-deficient mice has been shown to be only slightly delayed, although re-epithelialization at day 10 after wounding was 55% lower than in wild-type mice, whereas no delay was observed in *tpa*-deficient mice [16]. In addition to MMP-13, several MMPs including MMP-3 and -9, are expressed in murine leading edge kerationocytes [27]. Treatment with the broad-spectrum MP inhibitor Galardin, which is inhibiting a number of MMPs, ADAMS and ADAMTSs although with different IC_50_ values [35], [36] causes a delay in wound healing and effects on re-epithelialization very similar to those observed in *Plg*-deficient mice [19]. However, when *Plg*-deficient mice are treated with Galardin, healing is completely arrested, demonstrating a phenotypic overlap between the two families of matrix-degrading proteases in wound healing [19], [27]. The initial characteriations of *Mmp*-deficient mice, except for *Mmp14*, have only revealed subtle phenotypes during embryonic development and skin wound healing [for reviews see [6], [37]]. This could signify a non-vital involvement during embryonic development. It is also conceivable that the apparent dispensability of the MMPs can be explained by enzymatic functional overlap, compensatory upregulation or adaptive development, as has been exemplified by the generation of three individual MMP double-deficient mice [38]–[40] and *Plau*;*Plat* double-deficient mice [16], [21]. Since some MMPs have been shown to have overlapping substrates with the plasminogen activation system [9], [41] we have, by genetic means, generated mice deficient in *Mmp2* and *Plg* or *Mmp9* and *Plg* by intercrosses of double heterozygous *Mmp2;Plg or Mmp9;Plg* mice, respectively. This resulted in a genotypic distribution in accordance with the expected Mendelian distribution for *Mmp2;Plg* offspring whereas a non-Mendelian distributuion was observed for *Mmp9*;*Plg* offspring [42], [43]. Furthermore, we were not able to demonstrate any phenotypic overlap between MMP-2 and Plg or MMP-9 and Plg during wound healing [42], [43]. Thus, it still remains to be clarified which MMP, or which combinations of MMPs, are responsible for the phenotypic overlap with plasmin during wound healing. To address this further, we have generated mice that are double-deficient in *Mmp13* and *Plau*, the plasminogen activator most important for Plg activation in tissues. In a thoroughly characterized wound healing model [16], [27], where a 20 mm full thickness incisional skin wound is inflicted along the back midline of an anaesthetized mouse, we demonstrated a phenotypic overlap between MMP-13 and uPA, defined as an additional delay in wound healing in the double-deficient mice compared to the single-deficient mice. This finding was substantiated by an additional delay in wound healing in *Mmp13;Plg* double-deficient mice.

## Materials and Methods

### Mice

The generation of the *Mmp13*-, *Plg*- and *Plau*-deficient mice was previously described [18], [21], [39]. Both strains were backcrossed more than 18 generations to FVB/n. Heterozygous *Mmp13* and *Plau* mice were crossed to obtain an F1 generation of double heterozygous FVB-*Mmp-13^+/−^;Plau^+/−^* siblings. These mice were crossed to establish an F2 generation cohort from which four groups of siblings were selected: FVB-*Mmp13^+/+^;Plau^+/+^*, FVB-*Mmp13^−/−^;Plau^+/+^*, FVB-*Mmp13^+/+^;Plau^−/−^* and FVB-*Mmp13^−/−^;Plau^−/−^*. Similarly, heterozygous *Plg* gene-deficient mice backcrossed into an FVB/n background for 16 generations (Panum Institue, Copenhagen, Denmark) were mated with heterozygous *Mmp13* gene-deficient mice that were backcrossed into an FVB/n background for 9 generations to produce the F1 generation of double heterozygous FVB-*Mmp13^+/−^;Plg^+/−^* siblings. These F1 mice were crossed to produce an F2 generation from which four groups of siblings were selected for expermiments: FVB-*Mmp13^+/+^;Plg^+/+^*, FVB-*Mmp13^−/−^;Plg^+/+^*, FVB-*Mmp13^+/+^;Plg^−/−^* and FVB-*Mmp13^−/−^;Plg^−/−^*. Genotyping was performed on chromosomal DNA purified from tail tips by multiplex PCR using *Mmp13* [21], *Plau* [21] and *Plg* [18] primers. All animal experiments were conducted at The Department of Experimental Medicine, University of Copenhagen and Rigshospitalet, Copenhagen, Denmark, in accordance with institutional and national guidelines (permissions # 2007/561–1053). The review board at the Faculty of Health Science, University of Copenhagen (P06–114), approved this study. The mice were kept in single ventilated cages with free access to standard food and water. An observer unaware of the genotype of the mice performed all experimental evaluations.

### Skin Wounding

Six- to eight-week-old mice were anesthetized by intraperitoneal administration of a 1∶1 mixture of Dormicum (Roche, Basel, Switzerland) and Hypnorm (Janssen-Cilag Ltd, High Wycombe, UK) and shaved on the back before an incisional 20 mm-long full-thickness skin wound was made on the mice mid-dorsally with a scalpel. After wounding the mice were caged individually until termination of the experiment. The wounds were not dressed or sutured and they were observed and measured every second day until healing or isolation of tissue at the 14-day time point. Complete healing of the wound was defined by loss of wound crust. Previous studies revealed that this is an easy, robust, and reproducible method for analysis of overall healing of large incisional wound [16].

### Tissue Preparation

Mice were anesthetized as above followed by intracardial perfusion with 10 ml ice-cold phosphatebuffered saline (PBS) and 4% paraformaldehyde (PFA). The wound areas were removed down to the underlying fascia, bisected in the midtransversal plane and fixed in 4% PFA overnight at 4°C before they were embedded in paraffin. The sections were cut perpendicular to the longitudinal direction of the wounds. For histological analysis, a total of 77 mice were wounded, from which tissue was isolated for each of the four genotypes (wild-type, *Mmp13*-deficient, *Plau*-deficient and *Mmp13;Plau* double-deficient).

### Immunohistochemistry

Tissue sections were stained with the following antibodies: rabbit anti-mouse keratin 10 (1∶2000, BabCO, Richmond, CA, USA), rabbit anti-mouse keratin 14 (1∶2000, Covance, Berkeley, CA, USA), rat anti-mouse CD34 (1∶100, HyCult Biotechnology, Uden, The Netherlands) and rat anti-mouse F4/80 (BM8) (1∶200, eBioscience, San Diego, CA, USA). Tissue sections were deparaffinized in xylene and hydrated through graded ethanol/water dilutions. For collagen staining, sections were stained in Weigert's haematoxylin for 5 min, washed for 10 min in running tap water, stained for 10 min in 0.1% Picrosirius Red solution (Amplicon, Skovlunde, Denmark) followed by washes in two changes of acidified water (0.5% acetic acid) and rehydration in ethanol. For antibody staining, antigen retrieval was carried out by incubation in proteinase K for 15 min at 37°C (keratins 10/14, F4/80) or in citrate buffer pH 6.0 for 10 min at 98°C (CD34). After rinsing with water and TBS-T, endogenous peroxidase was quenched with 1% H_2_O_2_ for 15 min. For CD34 staining, endogenous biotin was blocked using a Biotin Blocking System (Dako, Glostrup, Denmark), followed by incubation with primary antibody over-night at 4°C, incubation with biotinconjugated rabbit anti-rat (CD34) and 30 min incubation with StreptABCopmplex/HRP (Dako). For F4/80 staining, sections were incubated with primary antibody for two hours at room temperature followed by secondary rabbit anti-rat IgG. For keratin 10/14, and F4/80 staining, sections were incubated in EnVision+ System Labelled Polymer-HRP Anti-rabbit (Dako) and all antibodies were detected using Vector® NovaRED™ substrate kit (Vector Laboratories, Burlingame, CA, USA). Controls without primary antibody were negative for unspecific staining.

### Computer-assisted morphometry

Keratinocyte migration of 14 days old skin wounds was measured by histological analysis of tissue sections immunohistochemically stained for cytokeratins as described [16]. Image analysis was performed using the Visiomorph software package (Visiopharm, Hørsholm, Denmark).

### Statistical Analysis

The GraphPad Prism software package (version 5.0) (GraphPad Software Inc., San Diego, CA, USA) was used for statistical analysis. Genotypic distribution was analyzed by *X* ^2^-test. Pre-specified tests of hypothesis comparing experimental groups were carried out using two-tailed t-tests. A p-value of 0.05 was set as the level of significance.

## Results

### Gestation is unaffected by simultaneous lack of *Mmp13* and either *Plau* or *Plg*

In order to examine and compare the effect of lack of either *Plau* or *Plg* combined with *Mmp13*- deficiency during gestation, we set out to generate *Mmp13;Plau* and *Mmp13;Plg* double-deficient mice. Mice lacking one or more *Mmp13* and *Plau* alleles were generated by inter-crosses of doubleheterozygous F1 parents. Analysis of the weaned F2 offspring revealed a Mendelian distribution (*X* ^2^-test, p = 0.656, Table 1). These results demonstrate that concomitant ablation of *Mmp13* and *Plau* did not affect the outcome of gestation and post-natal survival as determined by genotyping of the weaned offspring. In order to test whether complete ablation of *Plg* together with simultaneous lack of *Mmp13* is detrimental for gestation, the genotypes of weaned F2 offspring were analyzed revealing a Mendelian distribution (*X* ^2^-test, p = 0.208, Table 1). Taken together these results demonstrate that lack of *Mmp13* combined with lack of either *Plau* or *Plg* is dispensable for gestation.

**Table 1:** Genetic distribution of F2 offspring.

	*Mmp13;Plau F2*		*Mmp13;Plg F2*	
	Obs. (%)	Exp. (%)	Obs. (%)	Exp. (%)
**1. ++/++**	57	53	35	43
	(6.75)	(6.25)	(5.15)	(6.25)
**2. +−/++**	109	106	87	85
	(12.91)	(12.50)	(12.79)	(12.50)
**3. ++/−+**	93	106	105	85
	(11.02)	(12.50)	(15.44)	(12.50)
**4. +−/+−**	226	211	173	170
	(26.78)	(25.00)	(25.44)	(25.00)
**5. −−/++**	55	53	29	43
	(6.52)	(6.25)	(4.26)	(6.25)
**6. −−/−+**	112	106	80	85
	(13.27)	(12.50)	(11.76)	(12.50)
**7. ++/−−**	48	53	43	43
	(5.69)	(6.25)	(6.32)	(6.25)
**8. +−/−−**	101	106	83	85
	(11.97)	(12.50)	(12.20)	(12.50)
**9. −−/−−**	43	53	45	43
	(5.09)	(6.25)	(6.62)	(6.25)
**Total**	**844**	**844**	**680**	**680**
***X*** **^2^-test**	**p = 0.656**		**p = 0.208**	

The F2 offspring from interbreeding of double-heterozygous F1 breeding pairs (*Mmp13*;*Plau* and *Mmp13*;*Plg*) were genotyped at weaning. The numbers in brackets indicate the calculated incidence percentage.

### Phenotypic characterization of unchallenged *Mmp13;Plau* double-deficient mice

**Figure 1:**
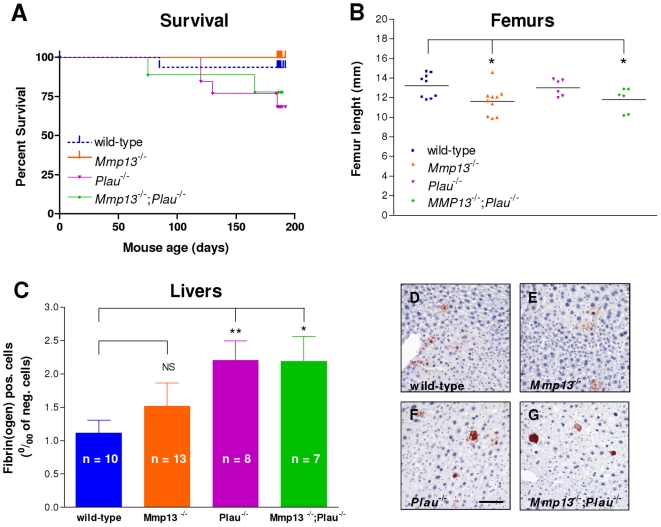
Survival, femur length and hepatic fibrin deposition in *Mmp13;Plau* double-deficient cohort mice. (**A**) Four genotype groups of siblings were selected for a survival cohort. (**B**) Femurs were removed from wild-type, *Mmp13*-deficient, *Plau*-deficient and *Mmp13;Plau* double-deficient mice at six months of age. Data are represented as scatter plots and mean values are depicted as horizontal lines. (**C–G**) Livers from mice were removed when mice were six months old, processed for immunohistochemistry and stained for fibrin. (**D–G**) Scale bar  = 0.2 mm. (**C**) The total area of liver cells positive for fibrin per mille of liver cells negative for fibrin was quantified. Bars represent the means ± SEM of each group. *p≤0.05; **p≤0.01.

To characterize *Mmp13;Plau* double-deficient mice with respect to development of spontaneous phenotypes during postnatal life, we selected four groups of offspring; 16 wild-type (9 males and 7 females), 14 *Mmp13*-deficient (8 males and 6 females), 13 *Plau*-deficient (7 males and 6 females) and 9 *Mmp13;Plau* double-deficient mice (6 males and 3 females). These mice were weighed and observed weekly until the age of six months for development of overt spontaneous phenotypes. Of these mice, 1 wild-type (female), no *Mmp13*-deficient, 4 *Plau*-deficient (1 male and 3 females) and 2 *Mmp13;Plau* double-deficient mice (2 females) died for unknown reasons (non-significant by Logrank test) (Figure 1A). The mice displayed no overt phenotypic differences and there was no difference in weight at any time point (data not shown). Additionally, like *Mmp13*- and *Plau*-deficient mice, both male and female *Mmp13;Plau* double-deficient mice were fertile and the females were capable of lactating and nursing their pups until weaning (data not shown).

*Mmp13* mRNA is expressed in hypertrophic chondrocytes and in the primary ossification center and mice lacking MMP-13 display an altered endochondral bone development which is resolved by 12 weeks of age [39]. Furthermore, *Mmp13*-deficient mice have increased tibia trabecular bone, a phenotype that progresses with age and is still apparent at 16 weeks of age, but resolved by one year of age [39]. We measured the length of femurs and tibiae at six months and found the femurs to be significantly shorter in *Mmp13*-deficient and *Mmp13;Plau* double-deficient mice compared to wild-type mice (p = 0.019 and 0.045), while there was no difference between wildtype and *Plau*-deficient mice (Figure 1B). There was no difference in tibia length between any of the four groups (data not shown). We also stained sections of the same femurs and tibiae with PicroSirius Red to look at trabecular bone, but found no differences between the four groups of offspring (data not shown).

Fibrin and fibronectin are substrates of plasmin, which is generated by uPA from the inactive precursor Plg [44], and mice deficient in uPA are known to develop spontaneous hepatic fibrin deposits [21]. *Mmp13* has been shown to be expressed by macrophages in murine livers where it is involved in mediating the regression of hepatic fibrosis [45] and MMP-13 activity against fibrinogen has been shown *in vitro* [46]. Thus, to examine a possible molecular functional overlap between uPA and MMP-13 on fibrinolysis, we measured the degree of fibrin accumulation in the mouse livers from all four groups of offspring. As expected we found fibrin plaque deposition to be significantly increased in *Plau*-deficient mice compared to wild-type mice (p = 0.005), and also in *Mmp13;Plau* double-deficient mice compared to wild-type mice (p = 0.014) (Figure 1, C–G). However, there was no difference between wild-type and *Mmp13*-deficient mice or *Plau*-deficient and *Mmp13;Plau* double-deficient mice, indicating that there is no molecular functional overlap between uPA and MMP-13 during postnatal fibrinolysis of the liver.

### Phenotypic overlap between MMP-13 and the plasminogen activation system in skin wound healing

**Figure 2:**
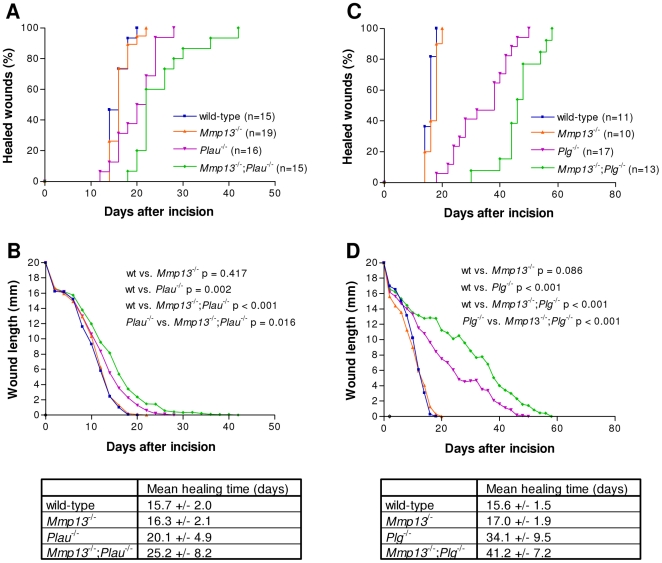
Time course of skin wound healing in *Mmp13;Plau* and *Mmp13;Plg* double-deficient mice. (**A**+**C**) The percentage fraction of mice with complete re-epithelialization is plotted vs. time after incision of 20 mm long wounds. (**B**+**D**) The average wound length is plotted vs. time after incision. Wound healing in *Mmp13*-deficient mice is indistinguishable from that in wild-type mice, while wound healing in both *Plau*- and *Plg*-deficient mice is significantly delayed compared to wildtype wound healing (p = 0.002 and p<0.001 in a two-tailed *t*-test). However, *Mmp13;Plau* and *Mmp13;Plg* double-deficient mice have an additional significant delay in wound healing compared to either *Plau*- or *Plg*-deficient mice (p = 0.016 and p<0.001 in a two-tailed t-test), indicating a phenotypic overlap between MMP-13 and the PA system.

Thus, observing no overt spontaneous phenotypes as a consequence of combined deficiency of *Mmp13* and *Plau*, we next initiated a wound healing study in which we inflicted standardized 20 mm-long full-thickness incisional skin wounds on wild-type (n = 15), *Mmp13*-deficient (n = 19), *Plau*-deficient (n = 16) and *Mmp13;Plau* double-deficient (n = 15) mice. The wounds were observed and lengths were measured every second day until healing. Complete healing of the wound was defined by loss of wound crust and closure of the incision interface with restoration of the epidermal covering. We found mice that are deficient in *Mmp13* to have a mean healing time indistinguishable from wild-type mice (16.3 versus 15.7 days), while *Plau*-deficient mice had a mean healing time that was delayed to 20.1 days (wild-type versus *Plau* ^−/−^; p = 0.002) (Figure 2, A and B). The mean healing time was increased to 25.2 days in mice that are deficient in both *Mmp13* and *Plau*, a significant delay compared to wildtype mice (p<0.001) and also to the *Mmp1*3 (p<0.001) and *Plau* (p = 0.016) single-deficient mice (Figure 2, A and B). The lack of an effect on wound healing in *Mmp13*-deficient mice has been demonstrated before and was expected [47]. We have previously examined the effect of *Plau*-deficiency in wound healing in a C57Bl6J background and found no effect on the mean healing time [16].

The phenotypic overlap between MMP-13 and the PA system was additionally substantiated by an independent wound healing study in wild-type (n = 11), *Mmp13*-deficient (n = 10), *Plg*-deficient (n = 17) and *Mmp13;Plg* double-deficient (n = 13) mice. As above, we found mice that are deficient in *Mmp13* to have a mean healing time indistinguishable from wild-type mice (17.0 versus 15.6 days), while *Plg*-deficient mice had a mean healing time that was delayed to 34.1 days (wild-type versus Plg−/−; p<0.001) (Figure 2, C and D). The mean healing time was increased to 41.2 days in mice deficient in both *Mmp13* and *Plg*, a significant delay compared to wild-type mice (p<0.001) and also to the *Mmp13* (p<0.001) and *Plg* (p = 0.001) single-deficient mice (Figure 2, C and D). Thus, during wound healing there is a phenotypic overlap between MMP-13 and uPA as well as MMP-13 and Plg.

### Histological examination of keratinocyte migration

**Figure 3:**
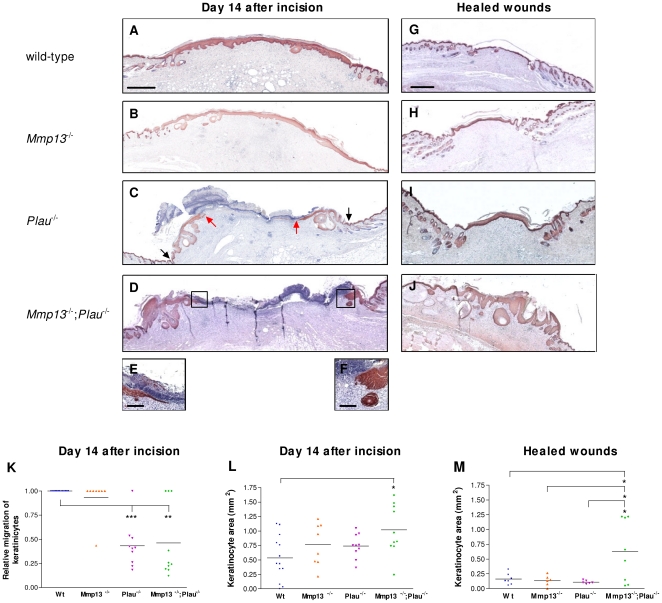
Kinetics of re-epithelialization in skin wounds in *Mmp13;Plau* double-deficient mice. Re-epithelialization of wounds is visualized by immunostaining of keratinocytes with anti-mouse keratins 10 and 14 in wild-type (**A**+**G**), *Mmp13*-deficient (**B**+**H**), *Plau*-deficient (**C**+**I**) and *Mmp13;Plau* double-deficient (**D**+**J**) mice. (**C**) **black arrows** mark the wound edge and **red arrows** point to the tip of the leading edge keratinocytes. Scale bar  = 0.5 mm, except for (**E**+**F**) (insets of **D** showing the leading edge keratinocytes) where scale bare  = 0.2 mm. (**K**) Quantitative evaluation of the relative migration distance of keratinocytes measured at day 14 after incision. With this method, complete re-epithelialization is scored as 1.0 and each data point refers to an individual wound. The mean values are depicted as horizontal lines. (**L**+**M**) Quantitative evaluation of the area of the multilayered epidermal layer measured from the wound edge to the leading edge kerationocytes by computerassisted morphometry. Differences in experimental groups are measured by a two-tailed unpaired t-test. *p≤0.05; **p≤0.01; ***p≤0.001).

Coverage of the wound with a new epidermis, a process called re-epithelialization, begins with keratinocyte hyperproliferation at the wound edges [25]. This is followed by migration of the keratinocytes, which is evident about 3–5 days after wounding [19]. We next examined histological sections of keratin-stained wounds removed 14 days after incision, when the wound healing curves for *Plau*- and *Mmp13;Plau*-double-deficient mice begin to separate, as well as two days after complete healing. After 14 days, the keratinocytes covered the wounds in 100% of the wildtype mice analyzed (Figure 3A), and only 12.5% of the wounds from the *Mmp13*-deficient mice were not covered (Figure 3B). Contrary to this, although the wound crust was clearly visible, 90% of the wounds from *Plau*-deficient and 70% from the *Mmp13;Plau*-double-deficient mice were not covered by keratinocytes at this time-point, demonstrating that the re-epithelialization is severely delayed (Figure 3, C and D). Apparently, this delay is not due to the migrating tips being blunt-ended, as they have been shown to be in wounds from mice deficient in *Plg* [19], [27] or in mice treated with the broad-spectrum MMP inhibitor Galardin [27]. The migrating tips in wounds from *Mmp13;Plau* double-deficient mice displayed both the phenotype characteristic of a wild-type wound where the leading-edge keratinocytes form a wedge-like structure (Figure 3E) as well as having blunt-ended tips (Figure 3F). This is true also for the *Plau*-deficient mice (data not shown).

We next quantified the re-epithelialization by measuring the relative migration of the keratinocytes, determined as the distance from the wound edge to the front of the leading-edge keratinocytes, divided by the distance between the two wound edges. At 14 days after wounding, we found the relative migration distances of the keratinocytes to be significantly lower in *Plau*- and *Mmp13;Plau* double-deficient wounds compared to wild-type wounds (p<0.001 and p = 0.002) (Figure 3K). However, there is no difference in relative migration between *Plau*- and *Mmp13;Plau* double-deficient wounds at this time-point.

When examining the wounds microscopically we noticed that in the *Mmp13;Plau* double-deficient wounds the epidermal layer was often multilayered with notable accumulation of keratinocytes protruding into the dermis below (Figure 3, D and J). Thus, we quantitated the keratin 10/14 immunoreactive area of the multilayered epidermis from the wound edge to the leading edge keratinocytes by computerassisted morphometry. We found that in the 14 day wounds the keratinocyte area in the *Mmp13;Plau* double-deficient wounds was significantly larger than in wild-type wounds (p = 0.010) (Figure 3L). Although notable, at this time point the increase was not significantly different from the areas measured in wounds single-deficient for either *Mmp13* or *Plau*. However, when measuring the keratinocyte area in healed wounds the difference between *Mmp13;Plau* double-deficient wounds and the other three genotypes became more evident. Healed wild-type, *Mmp13*- and *Plau*-deficient wounds were at this stage indistinguishable from each other with the epidermis forming a thin layer distinct from the underlying dermis (Figure 3, G–I). In contrast, the keratin 10/14 immunoreactive epidermal layers seen in the healed *Mmp13;Plau* double-deficient wounds were significantly larger and with notable protusion into the dermis compared to both wild-type (p = 0.037), *Mmp13*- (p = 0.043) and *Plau*-deficient wounds (p = 0.034) (Figure 3M). This finding demonstrates that the turnover of the multilayered epidermis, consisting of both basal and superbasel kerationcytes, is significantly delayed in *Mmp13;Plau* double-deficient healed wounds. This indicates that the phenotypic overlap between MMP-13 and uPA causing a delay in wound healing also causes a delayed turnover of the epidermis posthealing.

### Altered wound angiogenesis in *Mmp13;Plau* mice

**Figure 4:**
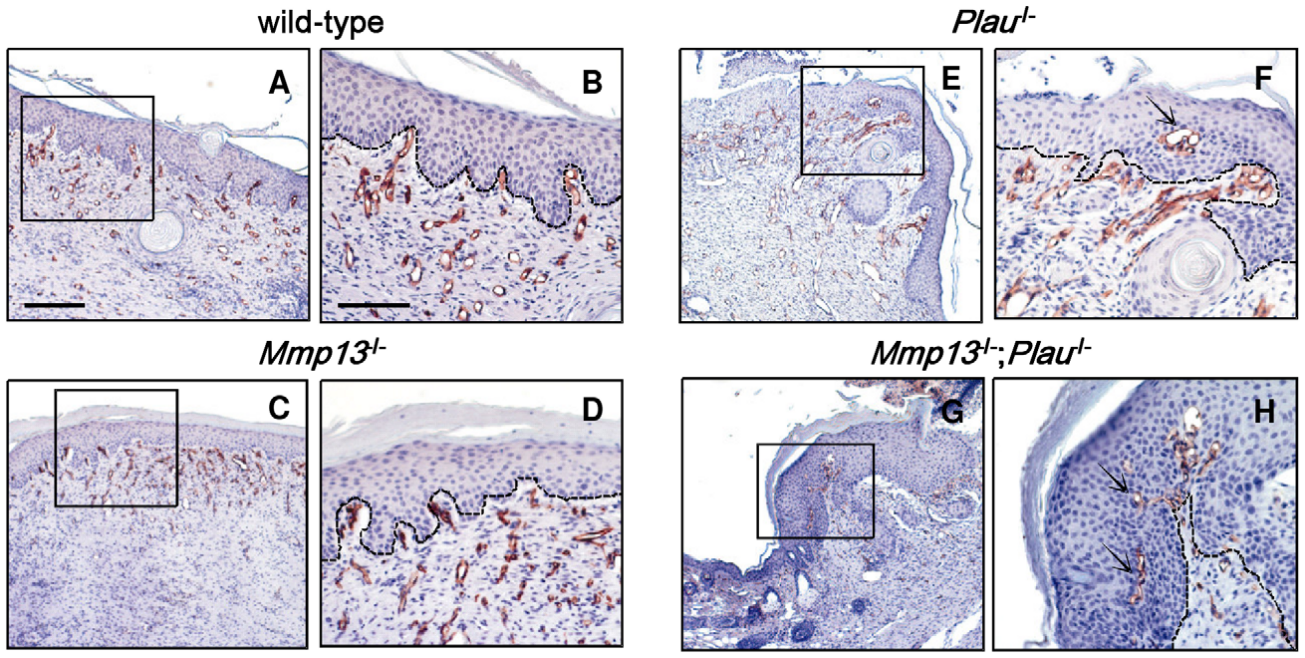
Angiogenesis in skin wounds in *Mmp13;Plau* double-deficient mice. Angiogenesis in wounds removed 14 days after incision is visualized by immunostaining of endothelial cells with antimouse CD34 in wild-type (**A**+**B**), *Mmp13*-deficient (**C**+**D**), *Plau*-deficient (**E**+**F**) and *Mmp13;Plau* double-deficient (**G**+**H**) mice. The box insets in (**A**, **C**, **E**+**G**) indicate the magnified views shown in (**B**, **D**, **F+H**). In (**F**+**H**) the **arrows** mark where the vessels protrude into the epidermal layer of the wounds. The scale bar in (**A**) = 0.2 mm and is representative also for (**C**, **E**+**G**). The scale bar in (**B**) = 0.1 mm and is representative also for (**D**, **F**+**H**).

During the phase of granulation tissue formation in wound healing, angiogenesis is stimulated by various growth factors, such as vascular endothelial growth factor (VEGF), hepatocyte growth factor (HGF), basic fibroblast growth factor (bFGF) and transforming growth factor β (TGF-β), derived from wound macrophages, keratinocytes and damaged endothelial cells [48], [49]. In addition, angiogenesis is dependent also on the proteolytic activity of plasmin and MMPs [7]. We therefore examined the effect of MMP-13 and uPA on angiogenesis in day 14 wounds by CD34 immunostaining. We found no apparent difference in the size or density of vessels when comparing wounds from wild-type, *Mmp13*-, *Plau*- or *Mmp13;Plau* double-deficient mice (Figure 4, A–H). The lack of effect on vessel size and density in *Mmp13*-deficient wounds is consistent with findings in another study on MMP-13 in wound healing [47], but in contrast to findings in a study where vessel density was found to be decreased [50]. Despite the lack of effect on vessel size and density in our study, we observed an altered CD34 expression pattern in wounds single-deficient for *Plau* or *Mmp13;Plau* double-deficient. Whereas the vessels in wild-type and *Mmp13*-deficient wounds were found exclusively in the dermis, concentrated at the interface between epidermis and dermis (Figure 4, A–D), the vessels protruded into the epidermal layer in the *Plau*- and *Mmp13;Plau* double-deficient wounds (Figure 4, E–H). Since this aberrant pattern was the same in *Plau*- compared to *Mmp13;Plau* double-deficient wounds, it suggests that the effect is due to the lack of uPA alone and does not explain the keratinocyte build-up and delayed wound healing observed in *Mmp13;Plau* double-deficient mice.

To determine the inflammatory response, wound tissue sections were also analyzed for infiltration of macrophages by F4/80 staining, but no difference between the four genotype groups was observed (data not shown). Additionally, immunohistochemical staining for laminin-5, which is expressed by leading edge keratinocytes and is a common substrate for both plasmin [51] and MMP-13 [52], revealed no aberrant pattern between any of the four genotype groups.

## Discussion

In this study we show that there is a phenotypic overlap between MMP-13 and the PA system in skin wound healing. We have previously reported that *Plg*-deficiency in mice results in a delayed wound healing. The primary reason for this delay is most likely a decreased ability of the keratinocytes to dissect their way proteolytically through the fibrin-rich extracellular matrix, which is substantiated by the fibrillar deposits seen in front of and below the epidermal outgrowth in *Plg*-deficient mice [27]. Nevertheless, the additional lack of fibrinogen does not completely rescue the impaired wound healing in *Plg*-deficient mice, indicating that plasmin has other substrates than fibrinogen during skin wound healing [19]. Moreover, mice treated with the broad-spectrum MP inhibitor Galardin have a delay in wound healing [19], [27]. Although excessive amounts of fibrin below and in front of the migrating keratinocytes are also seen in these mice [27], there is no significant difference in healing times between Galardin-treated wild-type mice and Galardin-treated fibrinogen deficient mice [19]. This indicates that reduced fibrinolysis is not the major cause of the delay in wound healing induced by Galardin.

Although wound healing is delayed in both *Plg*-deficient and Galardin-treated wild-type mice, wound healing is eventually achieved. However, when *Plg*-deficient mice are treated with Galardin healing is completely arrested, demonstrating that protease activity is essential for wound healing and that there is a phenotypic overlap between the PA- and the metalloprotease system, including MMPs, ADAM and ADAMTS in this process [27]. Both uPA and MMPs -3, -9 and –13, are expressed in the leading-edge keratinocytes of skin wounds, which may account for this very clear phenotypic overlap [27], [29]. Wound healing studies in mice deficient for MMPs -3, -9 and -13 have previously been performed. Just as incisional wound healing in *Mmp3*-deficient mice is not delayed compared to wild-type mice [53], there is either no or marginal (depending on the wound healing model used [50], [54]) delay in wound healing in *Mmp9*-deficient mice [43]. Regarding wound healing in *Mmp13*-deficient mice, two contrasting studies have been performed. In one study no differences in the re-epithelialization, inflammatory response, granulation tissue formation or angiogenesis is seen when comparing wild-type and *Mmp13*-deficient mice [47], while in the other study a delayed wound closure and a decreased vascular density is observed in *Mmp13*-deficient mice [50]. These discrepancies could be explained by the different genetic background of mouse strains used, or more likely by the differences in excisional wound sizes (4 mm versus 8 mm). To investigate a possible phenotypic overlap between MMP-13 and the PA system during normal physiology and wound healing, we have generated mice double-deficient for *Mmp13* and *Plau* as well as double-deficient for *Mmp13*- and *Plg*. The genotypic distribution of offspring from *Mmp13;Plau* and *Mmp13;Plg* heterozygous parents follows Mendelian distributions, suggesting that any possible molecular or phenotypic overlap between MMP-13 and uPA/Plg does not influence embryonic development and early post natal life. Additionally, the mice displayed no overt phenotypic differences.

To reveal a possible phenotypic overlap between MMP-13 and the PA system, we took advantage of a well-characterized skin wound healing model in which we made incisional 20 mm-long full-thickness skin wounds. We found that the combined deficiency in *Mmp13* and *Plau/Plg* results in a significant delay in wound healing compared to *Mmp13*, *Plau* or *Plg* single-deficiencies, demonstrating a phenotypic overlap between MMP-13 and uPA/Plg. We have previously provided evidence that plasmin cleavage of substrates other than fibrin(ogen) is rate-limiting for incisional skin wound healing [19]. Furthermore, the accumulation of fibrin(ogen) observed in front of keratinocytes in *Plg*-deficient mice is not observed in *Plau*-deficient wounds [16]. Thus, since we observe a phenotypic overlap, not only between MMP-13 and Plg but also between MMP-13 and uPA, we conclude that this is not exclusively due to impaired fibrin dissolution.

We observe no phenotypic difference between *Mmp13*-deficient and wild-type wounds suggesting that in the presence of uPA activity the lack of MMP-13 is compensated for. However, as detected by gelatin substrate zymography and fibrin overlay zymography no detectable compensatory upregulation of gelatinase activity or plasminogen activator activities was observed in the protease-deficient mice (data not shown).

One possible explanation for the additional delay in wound healing observed in *Mmp13;Plau* double-deficient mice is that the cleavage of one or more extracellular matrix components, which is critical for keratinocyte migration, can be achieved by either MMP-13 or uPA. We examined one such possible candidate, laminin-5, which is expressed by leading edge keratinocytes and is a common substrate for both plasmin [51] and MMP-13 [52]. However, we found no abberant expression pattern between the four groups of genotypes when performing immunohistochemical staining for laminin-5 in wounds. Thus, the existence, if any, of a mutual target for MMP-13 and uPA, which is functionally involved in keratinocyte migration still remains to be determined.

It would seem from our immunohistological examinations that the additional delay in wound healing observed in the *Mmp13;Plau* double-deficient mice is a result of hyperkeratosis, leading to a compressed build-up of keratinocytes under the skin surface. We examined the possible involvement of angiogenesis in this phenotype and found that in the *Plau*- and *Mmp13;Plau* double-deficient wounds the vessels protrude into the epidermal layer. Interestingly, this is never observed in wild-type and *Mmp13*-deficient wounds. It is well established that uPA is involved in the regulation of vascular remodeling and angiogenesis, not only in wound healing but also in tumors [55], [56]. The aberrant pattern of endothelial cells that we observe in the epidermis during wound healing suggests that the resulting effect of *Plau*-deficiency is not as simple as reduced vascular growth, since we observe no apparent difference in the size or density of vessels. It would seem that *Plau*-deficiency induces an imbalance between the various factors regulating angiogenesis, disrupting this normally very well controlled process. This aberrant pattern is not more pronounced in *Mmp13;Plau* double-deficient wounds compared to *Plau*-deficient wounds, and thus seems to be an effect related to *Plau*-deficiency alone. However, it is conceivable that the induction of angiogenesis in the epidermal layer induced by the lack of uPA, in combination with a separate proteolytic effect of MMP-13 causes the observed wound phenotype in the *Mmp13;Plau* double-deficient mice. According to such a scenario the observed phenotypic overlap between MMP-13 and the PA system during wound healing is not due to the existence of a mutual proteolytic substrate but rather two (or more) separate substrates with a cummulative effect. Thus, the function of MMP-13 alone is unmasked only when the PA system is simultaneously targeted.

It is very plausible that the phenotypic overlap between MMP-13 and uPA observed in the process of wound healing applies equally well to other normal or pathological tissue remodeling processes. In particular, there are many parallels between wound healing and cancer invasion [24], [25]. Epithelial cells in different types of human SCCs express both MMP-13 and uPA [12], [13], [22], [26], [30], and it would be interesting to examine this cancer type for a molecular and phenotypic overlap between the two proteases MMP-13 and uPA. Also interesting is the parallel to studies on MMP-13 and uPA in the murine MMTV-PyMT breast cancer model. Although MMP-13 and uPA are both highly expressed at the tumor-stroma border [57], [58], only deficiency in *Plau* causes a decreased lung metastasis volume [57], while *Mmp13*-deficiency has no effect [58]. In light of our results, it would be interesting to identify the effect of combined *Mmp13*- and *Plau*-deficiency in the MMTV-PyMT model. In conclusion, we have demonstrated a phenotypic overlap between MMP-13 and the PA system during remodeling of the skin, but not during gestation and postnatal development. It remains to be elucidated if this phenotypic overlap is also present in other tissue repair and remodeling events.
